# Influence of intravenous alteplase on endovascular treatment decision-making in acute ischemic stroke due to primary medium-vessel occlusion: a case-based survey study

**DOI:** 10.1136/neurintsurg-2021-017471

**Published:** 2021-05-25

**Authors:** Manon Kappelhof, Johanna Ospel, Nima Kashani, Petra Cimflova, Nishita Singh, Mohammed A Almekhlafi, Bijoy K Menon, Jens Fiehler, Michael Chen, Nobuyuki Sakai, Mayank Goyal

**Affiliations:** 1 Radiology and Nuclear Medicine, Amsterdam UMC, University of Amsterdam, Amsterdam, Netherlands; 2 Diagnostic Imaging, University of Calgary Cumming School of Medicine, Calgary, Alberta, Canada; 3 Radiology, University Hospital Basel, Basel, Switzerland; 4 Clinical Neurosciences, University of Calgary Cumming School of Medicine, Calgary, Alberta, Canada; 5 Department of Neuroradiology, University Medical Center Hamburg-Eppendorf, Hamburg, Hamburg, Germany; 6 Neurological Sciences, Rush University Medical Center, Chicago, Illinois, USA; 7 Neurosurgery, Kobe City Medical Center General Hospital, Kobe, Hyogo, Japan

**Keywords:** stroke, thrombolysis, thrombectomy

## Abstract

**Background:**

Intravenous alteplase is currently the only evidence-based treatment for medium-vessel occlusion stroke (MeVO; M2/3, A2/3, and P2/3 vessel segment occlusions), but due to its limited efficacy, endovascular treatment (EVT) is increasingly performed in these patients. In this case-based survey study, we examined the influence of intravenous alteplase treatment on physicians’ decision-making for EVT in primary MeVO stroke.

**Methods:**

In an international web-based survey among physicians involved in acute stroke care, participants provided their EVT decision for six quasi-identical fictional MeVO case scenarios (three with and without intravenous alteplase administered). Each scenario showed radiological images and clinical information in the form of a short case vignette. We compared EVT decisions (“immediate EVT”, “no EVT”, or “wait for alteplase effect” [in case scenarios with alteplase treatment only]) for case scenarios with and without alteplase treatment. Clustered multivariable logistic regression was performed to assess the effect of alteplase on treatment decision.

**Results:**

The survey was completed by 366 physicians from 44 countries, resulting in 2196 responses included in this study. In alteplase-treated cases, 641/1098 (58.4%) responses favored immediate EVT, (279/1098 [25.4%]) favored no EVT and 178/1098 (16.2%) opted to wait for alteplase effect. In non-alteplase-treated case scenarios, 846/1098 (78.7%) were in favor of and 252/1098 (21.3%) against EVT. Intravenous alteplase was associated with a lower chance of a decision in favor of immediate EVT (adjusted OR 0.38 [95%CI 0.31 to 0.46]).

**Conclusions:**

Intravenous alteplase is an important factor in EVT decision-making for MeVO stroke. However, even in alteplase-treated patients, more than half of the physicians decided to proceed with EVT without waiting for alteplase effect.

## Introduction

Intravenous alteplase followed by endovascular treatment (EVT) is the standard of care for acute ischemic stroke (AIS) due to large-vessel occlusion (LVO), in alteplase-eligible patients.^1^ With regard to AIS due to medium-vessel occlusion (MeVO), that is, M2/3, A2/3, and P2/3 occlusions, there is currently no randomized evidence proving the safety and efficacy of EVT. Thus, intravenous alteplase is the only treatment option with a level 1A guideline recommendation for MeVO patients, although many of them do not qualify for alteplase treatment due to their relatively mild symptoms (National Institutes of Health Stroke Scale (NIHSS) <6).^1–4^ However, alteplase efficacy in MeVO stroke is limited, with early complete recanalization following alteplase administration occurring in less than 50%.^5^ Given the overwhelming efficacy of EVT in LVO stroke^6^ and encouraging preliminary safety and efficacy data in the MeVO patient population,^7^ physicians are increasingly treating MeVO patients with EVT.^8–10^ A well-designed clinical trial to evaluate EVT in MeVO stroke is needed, but a logistical concern for such a trial includes understanding which factors may influence clinical equipoise for EVT. In this case-based survey study, we aimed to measure the degree of influence prior intravenous alteplase treatment would have on physicians’ EVT decision-making in MeVO stroke.

## Methods

The Conjoint Health Research Ethics Board of the University of Calgary approved this study (REB20-2086). An international, web-based, anonymous survey was conducted to explore current treatment practice and EVT decision-making in AIS due to MeVO among a broad, international audience of physicians involved in acute stroke care. Approximately 1400 stroke physicians of any specialty were invited to participate in the survey, based on their experience with EVT for AIS and their involvement in previous clinical trials on stroke. The online survey tool Qualtrics (Qualtrics.com) was used for survey distribution, data collection, encryption, and secure data storage. Access to the survey data was password-protected and granted to the study investigators only. Incomplete survey entries were excluded to avoid duplicate responses and incomplete data. Study data are available on reasonable request to the corresponding author.

### Survey design

Participants were shown 33 MeVO stroke case scenarios, each consisting of a set of imaging findings and accompanying clinical information in the form of short case vignettes that included a fictional patient’s age, baseline NIHSS, baseline ASPECTS, occlusion location of a primary isolated MeVO, and in some cases CT-perfusion-based core and penumbra volumes. Case vignettes were constructed based on combined data from multiple clinical cases and fictional data, and do not represent real patients. Participants were then asked whether they would offer EVT to the patient or not (binary response: yes/no). In case the vignette indicated that the patient received intravenous alteplase, a third response option was provided (“wait for alteplase effect”). This study analyzes response data from six case-scenarios: three in which patients received intravenous alteplase, and three quasi-identical scenarios, the only difference being that the case vignette indicated that the patient was ineligible for intravenous alteplase (table 1). Corresponding anonymous imaging findings as they were provided to the survey respondents are shown in online supplemental figures 1-3.

10.1136/neurintsurg-2021-017471.supp1 Supplementary data

**Table 1:** Fictional case scenarios used in this study

Scenario	Occlusion site	Age	Sex	NIHSS	Onset to CT time	ASPECTS	CTP imaging findings	Intravenous alteplase
1A	M2/3	86	Male	9	130 min	9	CTP core volume (rCBF <30%) 7 mL CTP penumbra volume: not provided	Yes
1B	M2/3	86	Male	9	130 min	10	CTP core volume (rCBF <30%) 7 mL CTP penumbra volume: not provided	Not eligible
2A	A3	79	Male	8	135 min	NA	CTP core volume (rCBF <30%) 9 mL CTP penumbra volume (Tmax >6 s) 30 mL	Yes
2B	A3	79	Male	8	135 min	NA	CTP core volume (rCBF <30%) 7 mL CTP penumbra volume (Tmax >6 s) 30 mL	Not eligible
3A	P2/3	52	Male	8	90 min	NA	CTP core volume (rCBF <30%) 4 mL CTP penumbra volume (Tmax >6 s) 24 mL	Yes
3B	P2/3	52	Male	8	90 min	NA	CTP core volume (rCBF <30%) 4 mL CTP penumbra volume (Tmax >6 s) 24 mL	Not eligible

*A3, third segment of the anterior cerebral artery; ASPECTS, Alberta Stroke Program Early CT Score for early ischemic changes on CT; CTP, CT perfusion; M2/3, second/third segment of the middle cerebral artery; NA, not applicable (ASPECTS only accounts for middle cerebral artery territory); P2/3, second/third segment of the posterior cerebral artery; rCBF, relative cerebral blood flow.

### Survey participants

Stroke physicians (neurologists, interventional neuroradiologists, neurologists, neurosurgeons, and other physicians directly involved in acute stroke care) were invited to participate in the survey, without restrictions for personal caseload, hospital setting, geographical location, experience, and level of training or career stage. Prior to answering the case scenarios, participants were asked to provide some basic information about their country of practice, years of experience in treating strokes, age range, specialty, and career stage.

### Statistical analysis

Participants’ baseline characteristics were summarized using descriptive statistics appropriate to the type of data. EVT decisions overall, with and without intravenous alteplase treatment were calculated and compared between physician specialties, geographic regions, and by occlusion location. Multivariable logistic regression with respondent identity and scenario as cluster variables was performed to assess the independent effect of intravenous alteplase on the decision to treat with EVT. Changes in treatment decision between scenarios with and without intravenous alteplase treatment decision were visualized with Sankey diagrams. Data analyses were performed in Stata 16.1. Figures were created with Microsoft Excel, version 16.45 and Microsoft PowerBI, version 2.88.1682.0.

## Results

A total number of 366 physicians from 44 countries completed the survey, resulting in 2196 responses for the six fictional case scenarios included in this study (n=1098 for scenarios with and without intravenous alteplase treatment each). In total, 217 survey entries were incomplete and excluded. Details on participants’ demographics are shown in online supplemental table 1.

### Intravenous alteplase and EVT decision

**Figure 1:**
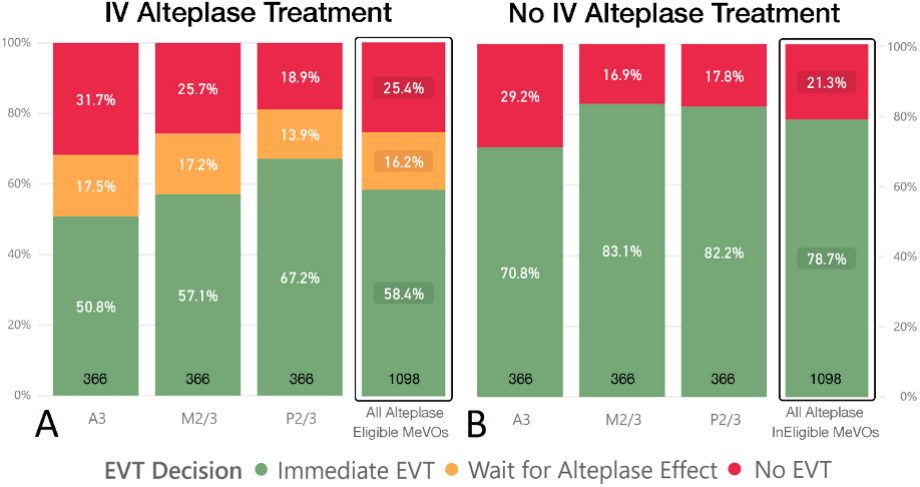
EVT decisions in scenarios with intravenous alteplase treatment (A) and without intravenous alteplase treatment (B), 1098 total responses in both groups. Note that “waiting for alteplase effect” was only an answer option in (A), that is, alteplase-eligible scenarios. A3, anterior cerebral artery third segment; EVT, endovascular treatment; IV, intravenous; M2/3, middle cerebral artery second/third segment; MeVOs, medium-vessel occlusions; P2/3, posterior cerebral artery second/third segment.

For case scenarios where intravenous alteplase was administered, 641/1098 (58.4%) responses were in favor of immediate EVT, and “no EVT” was the second most common answer (279/1098 responses [25.4%]), while only 178/1098 (16.2%) responses were in favor of waiting for intravenous alteplase effect, as shown in figure 1A. When the patient described in the scenario was not eligible for intravenous alteplase treatment before EVT, immediate EVT was favored by 846/1098 (78.7%), see figure 1B. When stratifying responses by occlusion site, decision rates in favor of immediate EVT were lowest in A3 occlusion scenarios and highest in P2/3 occlusion scenarios, both with and without alteplase treatment. In multivariable clustered logistic regression, intravenous alteplase treatment (adjusted OR 0.38; 95% CI 0.31 to 0.46) was strongly negatively associated with the decision to proceed with immediate EVT.

**Figure 2:**
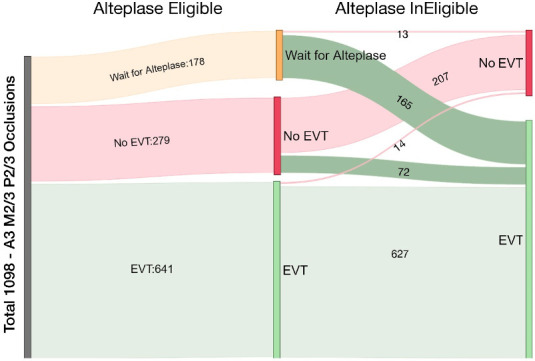
Changes in EVT decision between scenarios with and without intravenous alteplase treatment. The vertical gray bar on the left represents the total number of responses. The vertical bars in the middle of the diagram show EVT decisions for alteplase-eligible case scenarios (either “immediate EVT”, “no EVT”, or “wait for alteplase effect”). Vertical bars on the right show EVT decisions for alteplase-ineligible case scenarios. The width of the streams is proportional to the number of responses. Note that respondents who initially decided to wait for alteplase effect mostly decided to proceed with EVT in alteplase-ineligible case scenarios. A3, anterior cerebral artery third segment; EVT, endovascular treatment; M2/3, middle cerebral artery second/third segment; P2/3, posterior cerebral artery second/third segment.

Changes in EVT decision between alteplase-eligible and alteplase-ineligible patients are visualized in figure 2. Among physicians who decided to wait for alteplase treatment effect in eligible patients (n=63/366 [17.2%] in M2/3 occlusions, n=64/366 [17.5%] in A3 occlusions, n=51/366 [13.9%] in P2/3 occlusions), most decided to proceed with immediate EVT in case the described patient was not eligible for alteplase treatment (n=59/63 [93.7%] in M2/3 occlusions; n=57/64 [89.1%] in A3 occlusions; n=49/51 [96.1%] in A3 occlusions).

### Participant’s characteristics and EVT decision

**Figure 3:**
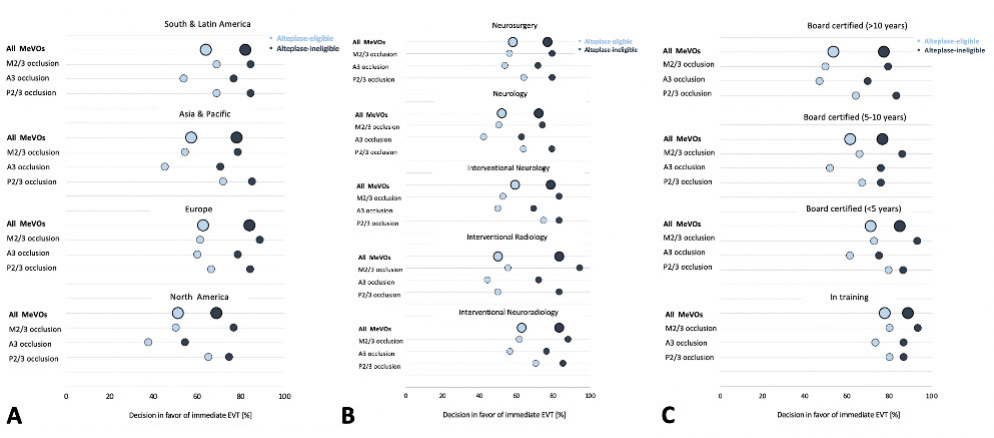
Overall and occlusion location-specific decisions in favor of immediate EVT by respondents’ region of practice (A), specialty (B), and career stage (C). Light blue circles indicate decision rates in favor of immediate EVT for alteplase-eligible scenarios and dark blue circles indicate decision rates in favor of immediate EVT for alteplase-ineligible scenarios. Only geographical regions and specialties with >10 responses per occlusion location are shown. A3, anterior cerebral artery third segment; EVT, endovascular treatment; M2/3, middle cerebral artery second/third segment; MeVOs, medium-vessel occlusions; P2/3, posterior cerebral artery second/third segment.

A comparison of overall and occlusion location-specific EVT decisions between geographic regions, specialties, and career stages is shown in figure 3. Physicians from Europe decided most often in favor of immediate EVT (337/537 [62.8%] with and 451/537 [84.0%] without intravenous alteplase treatment (figure 3A), although rates were overall similar across geographic regions. Immediate EVT was consistently favored more often in alteplase-ineligible case scenarios, and immediate EVT decision rates were lowest for A3 occlusions in all regions. Figure 3B shows overall and occlusion location-specific EVT decision rates by physician specialty. Interventional neuroradiologists and neurosurgeons showed the highest decision rates in favor of immediate EVT, while neurologists favored immediate EVT least frequently. Across all specialties, EVT decision rates for A3 occlusions were lowest compared with M2/3 and P2/3 occlusions, independent of the patient’s alteplase eligibility status. With increasing physician experience, decision rates in favor of immediate EVT with and without alteplase constantly decreased, and the “gap” between decision rates for alteplase-eligible and alteplase-ineligible scenarios increased (figure 3C).

## Discussion

In this study, intravenous alteplase treatment was significantly associated with decreased chances of a decision in favor of immediate EVT in MeVO stroke, but even in alteplase-eligible patients, the majority of physicians decided to proceed with EVT without waiting for an alteplase treatment effect. Decision rates in favor of immediate EVT were highest among junior physicians, physicians from Europe, and those trained in interventional neuroradiology and neurosurgery.

Previous studies report higher rates of early recanalization with a slight, although non-significant, improvement in clinical outcomes following MeVO stroke with intravenous alteplase administration.^5^ Our results clearly reflect this alteplase treatment effect, with significantly higher EVT decision rates in alteplase-ineligible patients (79% vs 58%). Furthermore, one out of six physicians stated that they would wait to see whether the patient shows clinical improvement after alteplase before making a decision on EVT. However, probably owing to the rather small effect of alteplase on functional outcome in previous studies, more than half of the survey participants decided to immediately proceed with EVT even in patients who received intravenous alteplase. Out of those who decided to wait for an alteplase treatment effect, the vast majority opted for immediate EVT if alteplase was contraindicated.

However, all described patients in our survey had an NIHSS score of 8 or 9. Guidelines advise EVT in LVO strokes from NIHSS 6: strokes with less neurological deficits are often considered mild. For patients with mild strokes, which account for a relatively large proportion of MeVO patients due to the more distal occlusion location, intravenous alteplase was shown to improve functional independence rates,^11^ whereas an additional benefit of EVT in this patient group has not been proven.^12 13^ As such, more physicians might have preferred to wait for the effect of administered alteplase if patients had lower NIHSS scores. In addition, less severe neurologic deficits in MeVO stroke may affect patient triaging, in turn affecting workflow times and alteplase efficacy.^14^ In LVO strokes, the role of alteplase in patients presenting directly to an EVT-performing center seems limited, since direct EVT and alteplase followed by EVT give very similar outcomes.^15^

Procedural complications of EVT may form a drawback for physicians treating MeVO strokes. Important complications after EVT include intracranial hemorrhage and vessel dissection.^6^ These risks may be increased in MeVO strokes, since the target vessels are smaller and more distally located. However, new techniques, smaller devices, and more flexible catheters specifically designed for distal thrombectomy have improved procedural safety.^16–18^

EVT decision rates in this study differed based on occlusion location. In scenarios with A2/3 occlusion, immediate EVT was favored only by 51% (alteplase-eligible patients)/71% (alteplase-ineligible patients) of respondents, while these numbers were much higher for scenarios with M2/3 occlusion (57%/83%) and P2/3 occlusion (82/67%). The relatively high willingness to proceed with immediate EVT in P2/3 MeVOs could potentially reflect the lack of high-level evidence for IV thrombolysis in posterior circulation MeVOs,^19^ though one could argue that this applies to EVT as well. In fact, the safety and efficacy of EVT has not even been formally proven for LVOs in the posterior circulation,^20^ and there is complete paucity of high-level evidence on EVT for MeVOs in posterior circulation. The relatively young patient age (52 years), short onset to CT time (90 min), and small ischemic core volume (4 mL) in the posterior circulation occlusion scenario probably also influenced participants’ treatment decisions. The reason for the relatively low EVT decision rates in the scenarios with A3 occlusion remains unclear. They could be partly related to the fact that the deficits in isolated, more distal anterior cerebral artery occlusions can be relatively subtle and are often not well reflected in the NIHSS. It is however important to note that this impression is often deceptive: common sequelae of anterior cerebral artery strokes such as memory impairment, emotional lability, and isolated sensory deficits are often highly disabling, although they might not immediately become obvious in a focused neurological examination.^21^ The same applies to the validity of the NIHSS in PCA strokes: P2/P3 occlusions often present with lower NIHSS scores, possibly because the NIHSS does not optimally reflect the typical posterior circulation stroke symptoms.^10^ Therefore, we included detailed descriptions of the neurological loss of function for each case scenario, rather than only an NIHSS value (see online supplemental figures 1-3).

We observed an inverse relationship between physician experience and the willingness to proceed with immediate EVT, with physicians in training opting most often for immediate EVT, and board-certified physicians with >10 years of experience deciding least often in favor of immediate EVT. Furthermore, the “gap” between EVT decisions for alteplase-eligible and -ineligible scenarios decreased in less experienced physicians. This may indicate that younger physicians, having seen more of the rise and successes of EVT after the trials of 2015/2016,^22^ and less of the negative trials in the preceding years,^23^ have more positive expectations of EVT outcomes. For alteplase, the same effect may be true for physicians who saw the successes after the trials in the late 1990s.^24–26^ Of note, our case-scenarios specifically referred to intravenous alteplase, which is currently considered the standard of care. Whether physicians’ decisions would have been different with an alternative thrombolytic agent such as tenecteplase, which shows improved recanalization rates than alteplase, remains unclear at this point.^27 28^

With regard to physician specialties, the highest willingness to proceed with immediate EVT was seen in interventional neuroradiologists and neurosurgeons. A similar effect as for the early vs late-career differences may be present, where neurologists may have been more involved in the alteplase trials, whereas interventionalists were logically only involved in the EVT trials. Confidence and experience in EVT efficacy and especially safety may play a role in this difference as well. Overall however, differences were small, and decision rates were largely similar between specialties.

When stratifying by geographic regions, European physicians showed the highest decision rates in favor of immediate EVT, closely followed by South/Latin America. The reasons for these geography-specific findings are complex and might be among other reasons related to local healthcare policy, guidelines, and medicolegal environment. Another potential explanation is the frequent use of general anesthesia for EVT in Europe.^29^ General anesthesia completely eliminates patient movement and might benefit the safety of MeVO EVT, where vessels are much smaller and more prone to injury than in LVO stroke.

### Limitations

This study has limitations. First, physicians were invited to this survey based on institutional networks and collaborations, and some geographic regions and specialties were under-represented. Thus, the results may not be representative of the entire stroke community. We collected no data on the number and characteristics of physicians that did not accept the survey invitation. Second, survey data are cross-sectional and thus do not reflect changes in EVT treatment decisions over time, which are invariably bound to happen as existing EVT technologies evolve and new devices are developed. Third, since vascular anatomy plays a key role in EVT decision-making, participants might have been influenced by the specific images provided in each case scenario, and their decision may reflect decision-making for this particular case rather than decision-making for MeVOs in general. We only included a limited number of occlusion locations: for example, we did not include a case with an A3 occlusion, which may have caused underrepresentation of some MeVO patient subgroups with certain occlusion locations in our study findings. Fourth, owing to the high number of responses, almost all findings in this study, for example subspecialty-related differences in EVT decision rates, were statistically significant but the absolute difference was often small and perhaps of no clinical relevance. We therefore deliberately refrained from reporting P-values for these differences. Lastly, simplified clinical case scenarios as they were used in this study can never capture the many dimensions of clinical decision-making in routine practice. For example, all case scenarios had an NIHSS of 8 or 9. A scenario with NIHSS <6, below the current cut-off for guideline-advised EVT for LVO strokes, would have given useful additional information – although the NIHSS may underrepresent in posterior and anterior circulation strokes, as described above. In addition, EVT preference rates in clinical practice may vary between patients with occlusions of the M2 vs M3 segment, or P2 vs P3, or A2 vs A3, and many other individual patient factors may make the decision to treat with EVT markedly more complex than the dichotomization we describe.

## Conclusions

Intravenous alteplase treatment was an important factor in EVT decision-making for MeVO stroke in this study, but even in alteplase-eligible patients, more than half of the physicians decided to proceed with EVT immediately, without waiting for alteplase effect.

## Data Availability

Data are available from the corresponding author upon reasonable request.
