# Supplementary material for: Influence of intravenous alteplase on endovascular treatment decision-making in acute ischemic stroke due to primary medium-vessel occlusion: a case-based survey study
Source: J Neurointerv Surg. 2021 May 25;14(5):439–43. doi: 10.1136/neurintsurg-2021-017471 (PMC9016248; doi:10.1136/neurintsurg-2021-017471)
Supplement: Supplementary data [file neurintsurg-2021-017471supp001.pdf]

SUPPLEMENTARY MATERIAL

Supplementary Figure 1: Images shown in the M2/3 occlusion case-scenarios. Multiphase CTA with right MeVO (medium vessel occlusion) - M2/M3 MCA (pink arrowheads). Delayed washout in the third phase (III). Accompanying case descriptions:

<u>1A)</u> 86-year-old male, otherwise healthy, NIHSS 9 with left-sided weakness, facial palsy, and hemineglect. Onset-to-CT time 2 hours 10 minutes. ASPECTS 9, core (CBF <30%) volume is 7 ml. Patient received tPA 10 minutes ago.

<u>1B)</u> 86-year-old male, otherwise healthy, NIHSS 9 with left-sided hemiplegia and facial palsy. Onset-to-CT time 2 hours 10 minutes. ASPECTS 10, core (CBF <30%) volume is 7 ml. Patient is not eligible for tPA.

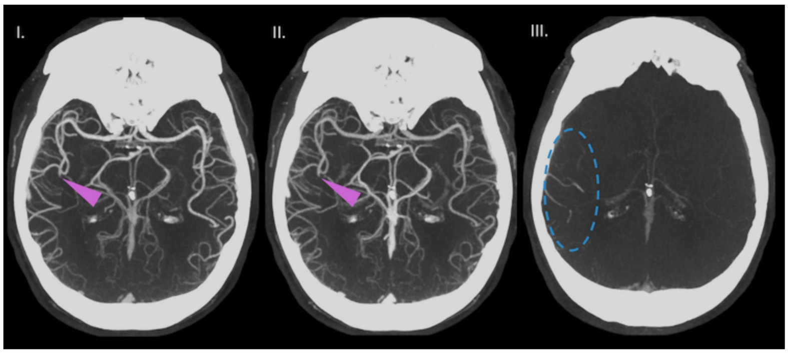

Supplementary Figure 2: Images shown in the A3 occlusion case-scenarios. Right MeVO - A3 ACA (pink arrowhead). Red line on the sagittal projection represents the course of the missing artery. Turquoise line represents the course of the terminal ICA and the ACA origin. Accompanying case descriptions:

<u>2A)</u> 79-year-old male, otherwise healthy, NIHSS 8 with left leg and arm weakness, left-sided sensory loss and left facial palsy. Onset-to-CT time 2 hours 15 minutes. Core (CBF <30%) volume is 9 ml, penumbra (Tmax >6s) 30 ml. Patient received tPA.

<u>2B)</u> 79-year-old male, otherwise healthy, NIHSS 8 with left leg and arm weakness, left-sided sensory loss and left facial palsy. Onset-to-CT time 2 hours 15 minutes. Core (CBF <30%) volume is 9 ml, penumbra (Tmax >6s) 30 ml. Patient is not eligible for tPA.

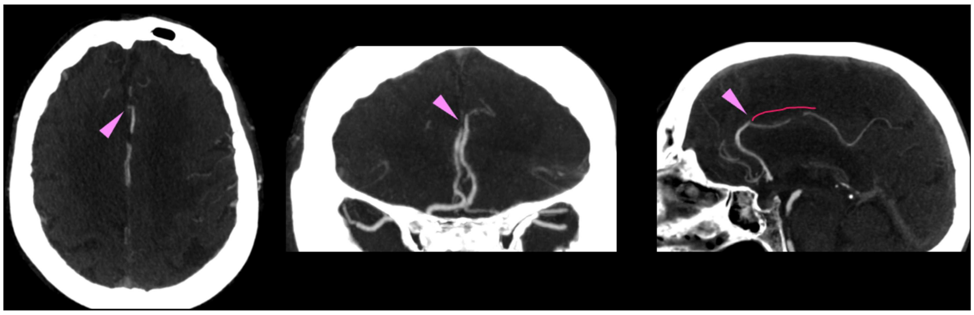

Supplementary Figure 3: Images shown in the P2/3 occlusion case-scenarios. CTA shows a right MeVO - P2/P3 PCA. Accompanying case descriptions:

3A) 52-year-old male, truck driver, NIHSS 8 with mild left-sided weakness, left homonymous hemianopia, confusion, not following commands. Onset-to-CT time 1 hour 30 minutes. Core (CBF <30%) volume is 4 ml, penumbra (Tmax >6s) 24ml. Patient received tPA 10 minutes ago.

3B) 52-year-old male, truck driver, NIHSS 3 with mild left arm weakness and left homonymous hemianopia. Onset-to-CT time 1 hour 30 minutes. Core (CBF <30%) volume is 4 ml, penumbra (Tmax >6s) 24ml. Patient is not eligible for tPA.

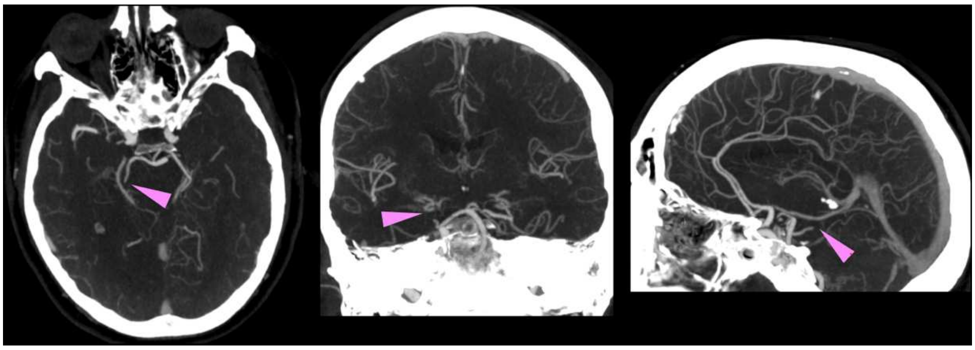

Supplementary Table 1: Demographics of physicians (N=366) participating in the survey. *IQR, interquartile range; EVT, endovascular treatment.*

**Physician demographics (N=366)**	**Value**
Specialty – *n (%)*	
Interventional Neuroradiologist	170 (46.5)
Interventional Radiologists	18 (4.9)
Interventional Neurologist	36 (9.8)
Neurologist	97 (26.5)
Neurosurgeons	39 (10.7)
Geographic region – *n (%)*	
North America	95 (26.2)
Europe	179 (49.5)
Asia & Pacific & Africa	75 (20.7)
South America	13 (3.6)
Hospital Setting – *n (%)*	
Teaching	337 (92.1)
Non-teaching	29 (7.9)
Physician gender – *n (%)*	
Female	56 (15.3)
Male	308 (84.1)
Do not wish to declare	2 (0.6)
Age - *n (%)*	
Under 30 years	5 (1.4)
31 – 40 years	96 (26.2)
41 – 50 years	151 (41.3)
51 – 60 years	80 (21.9)
Over 60 years	34 (8.3)
Experience in vascular neuro-interventions – *n (%)*	
0 - 5 years	51 (13.9)
5 -10 years	81 (22.1)
10 – 15 years	77 (21.0)
15 – 20 years	53 (14.5)
More than 20 years	52 (14.2)
Not applicable	52 (14.2)
Range of annual center thrombectomy volume – *median*	100-200
Range of annual personal thrombectomy volume – *median*	10-50
Number of interventionalists – *median (IQR)*	3 (3-5)
Availability of 24/7 coverage for EVT – *n (%)*	
Yes	332 (90.7)
No	34 (9.3)

Supplementary Table 2: Detailed country of practice of physicians (N=366) participating in the survey.

Respondent country	Frequency (n=)	%
Armenia	1	0.27
Australia	6	1.64
Austria	1	0.27
Belgium	7	1.91
Brazil	8	2.19
Canada	39	10.66
China	10	2.73
Colombia	1	0.27
Croatia	1	0.27
Czech Republic	5	1.37
Denmark	8	2.19
France	6	1.64
Georgia	1	0.27
Germany	34	9.29
Greece	1	0.27
India	15	4.10
Iran	1	0.27
Israel	1	0.27
Italy	16	4.37
Japan	11	3.01
Kazakhstan	1	0.27
Kuwait	1	0.27
Latvia	1	0.27
Mexico	3	0.82
Netherlands	16	4.37
New Zealand	3	0.82
Norway	7	1.91
Pakistan	1	0.27
Peru	1	0.27
Poland	4	1.09
Portugal	7	1.91
Republic of Korea	24	6.56
Romania	2	0.55
Russian Federation	7	1.91
Serbia	2	0.55
Slovakia	1	0.27
South Africa	2	0.55
Spain	22	6.01
Sweden	2	0.55
Switzerland	12	3.28
Turkey	3	0.82
United Kingdom	11	3.01
USA	56	15.30
Vietnam	4	1.09
**Total**	**366**	**100.00**
